# HIV, Sexually Transmitted Diseases, Tuberculosis, and Malaria: Resurgence and Response^1^

**DOI:** 10.3201/eid1011.040797_04

**Published:** 2004-11

**Authors:** Scott Holmberg, Scott McNabb, Sevgi Aral

**Affiliations:** * Centers for Disease Control and Prevention, Atlanta, Georgia, USA

**Keywords:** HIV, Sexually Transmitted Diseases, Tuberculosis, Malaria

Approximately 40% of the world's population is at risk for malaria; 300–500 million cases and 1–2 million deaths occur from this disease each year. Malaria control includes case management; prevention efforts; environmental management; and education of patients, healthcare workers, and the private sector. Because antimalarial drug resistance has emerged as a major problem, especially in sub-Saharan Africa, since 2001, the World Health Organization has recommended combining antimalarial drugs for first-line treatment, preferably artemisinin-based combination therapies. Global supply and production of such therapies are insufficient to meet demand; treating 400–850 million Africans who need it could require U.S.$3.4 billion. Still, the potential benefits of even suboptimal use has led to an unprecedented international effort to increase production and availability, decrease cost, and shift to better case-management policies.

Tuberculosis (TB) is responsible for 8.8 million cases and 2 million deaths each year, with marked increases in Africa, East Europe, and Asia. Although TB drugs are inexpensive, the slow metabolism of the disease makes treatments shorter than 3 to 4 months unlikely in the near future. In the United States, cases in immigrants have remained relatively stable in recent years, but the following barriers to TB control remain: unsettled status and mobility of infected persons, differences in epidemiology, drug resistance, coinfection with HIV, complexity and costs of screening and treatment, and social stigmatization. To combat TB locally and globally, three components are necessary: technical rigor, sustained funding, and good management. These approaches include the directly observed therapy short course (DOTS) strategy, which is highly effective and increasingly used worldwide.

After a decade's decline, U.S. rates of primary and secondary syphilis have increased among men in both 2001 and 2002. On the basis of male/female rate ratios and data collected during outbreak investigations, much of the increase can be attributed to men who have sex with men. A high rate of HIV coinfection—from 40% to 60% of the men acquiring syphilis—has been reported in these outbreaks, which are also characterized by exposure to multiple partners, prevalent substance abuse, and meeting partners in a variety of venues, including the Internet. In response, health departments are increasing provider education, strengthening community outreach, and making syphilis testing and treatment available in venues outside of traditional sexually transmitted disease (STD) clinics. Many factors contribute to the observed increases in syphilis, including prevention burnout and complacency about HIV/AIDS in light of improved treatments.

On every comparative measure of HIV and AIDS prevalence and incidence in the United States, the South shows the greatest increases. Several specific studies reinforce the urgency of the HIV epidemic in the South. HIV is now being acquired locally (rather than from visits to big cities) and is highly associated with substance abuse, mainly alcohol and crack, and with sex-trading. Even among those in HIV care, 40% were still sexually active in the previous month; 10% had visited an STD clinic in the previous year; 27% reported that they were not told where to get health care when they first discovered they are HIV-infected; 38% had no medical insurance (16% had lost it upon becoming HIV-infected); and 13% did not seek some care because they could not afford it. In one study in Alabama, HIV therapy seemed to lead to significantly diminished likelihood of gay men's using condoms consistently with their sex partners. There are many obstacles to better access to and use of health care for HIV in the South.

